# QTL Analysis of Transgressive Nematode Resistance in Tetraploid Cotton Reveals Complex Interactions in Chromosome 11 Regions

**DOI:** 10.3389/fpls.2017.01979

**Published:** 2017-11-20

**Authors:** Congli Wang, Mauricio Ulloa, Tra T. Duong, Philip A. Roberts

**Affiliations:** ^1^Department of Nematology, University of California, Riverside, Riverside, CA, United States; ^2^Key Laboratory of Mollisols Agroecology, Northeast Institute of Geography and Agroecology, Chinese Academy of Sciences, Harbin, China; ^3^Plant Stress and Germplasm Development Research, PA, CSRL, USDA-ARS, Lubbock, TX, United States

**Keywords:** root-knot nematodes (RKN), *Meloidogyne incognita*, *Gossypium* spp., upland, Pima, allele interactions

## Abstract

Transgressive segregation in cotton (*Gossypium* spp.) provides an important approach to enhance resistance to the major pest root-knot nematode (RKN) *Meloidogyne incognita*. Our previous studies reported transgressive RKN resistance in an intraspecific *Gossypium hirsutum* resistant NemX × susceptible SJ-2 recombinant inbred line (RIL) population and early generations of interspecific cross *Gossypium barbadense* (susceptible Pima S-7) × *G. hirsutum* (NemX). However, the underlying functional mechanisms for this phenomenon are not known. In this study, the region of RKN resistance gene *rkn1* on chromosome (Chr) 11 and its homoeologous Chr 21 was fine mapped with *G. raimondii* D_5_ genome reference sequence. Transgressive resistance was found in the later generation of a new RIL population F_2:7_ (Pima S-7 × NemX) and one interspecific F_2_ (susceptible Pima S-7 × susceptible SJ-2). QTL analysis revealed similar contributions to root-galling and egg-production resistance phenotypes associated with SSR marker CIR316 linked to resistance gene *rkn1* in NemX on Chr 11 in all seven populations analyzed. In testcross NemX × F_1_ (Pima S-7 × SJ-2) marker allele CIR069-271 from Pima S-7 linked to CIR316 contributed 63% of resistance to galling phenotype in the presence of *rkn1*. Similarly, in RIL population F_2:8_ (NemX × SJ-2), SJ-2 markers closely linked to CIR316 contributed up to 82% of resistance to root-galling. These results were confirmed in BC_1_F_1_ SJ-2 × F_1_ (NemX × SJ-2), F_2_ (NemX × SJ-2), and F_2_ (Pima S-7 × SJ-2) populations in which up to 44, 36, and 15% contribution in resistance to galling was found, respectively. Transgressive segregation for resistance was universal in all intra- and inter-specific populations, although stronger transgressive resistance occurred in later than in early generations in the intraspecific cross compared with the interspecific cross. Transgressive effects on progeny from susceptible parents are possibly provided in the *rkn1* resistance region of chromosome 11 by tandemly arrayed allele (TAA) or gene (TAG) interactions contributing to transgressive resistance. Complex TAA and TAG recombination and interactions in the *rkn1* resistance region provide three genes and a model to study disease and transgressive resistance in polyploid plants, and novel genotypes for plant breeding.

## Introduction

Host-plant resistance is a highly effective strategy to manage root-knot nematode (RKN, *Meloidogyne* spp.) damage in crops. The RKN *Meloidogyne incognita* is one of the most important pests of cotton (Goodell and Montez, 1994), and effective resistance is available for improving cotton cultivar performance (Starr et al., 2010). In addition, transgressive segregation is one of the approaches to enhance resistance, in which segregating hybrids exhibit extreme or novel phenotypes compared to the phenotypes of parental lines (Rieseberg et al., 1999, 2003). Improved resistance traits generated by transgressive segregation in progenies derived from interspecific and intraspecific crosses have been reported (Cherif and Harrabi, 1993; Zhang et al., 2001; Imtiaz et al., 2003a,b; Navabi et al., 2004; Bell and Travis, 2005; Zhao et al., 2005; Staal et al., 2006; Aghnoum and Niks, 2011). Transgressive segregation provides one of the major selection sources for enhanced resistance to RKN in cotton (*Gossypium hirsutum* L.) (Shepherd, 1974; Wang et al., 2006a; Wang C. et al., 2008, 2012; Ulloa et al., 2016). Other cotton-pathogen systems also displayed transgressive resistance, such as Fusarium wilt (Wang and Roberts, 2006a; Ulloa et al., 2011, 2013, 2016), Verticillium wilt (Bolek et al., 2005; Wang H. M. et al., 2008), and bacterial blight (Bayles et al., 2005).

Three major germplasm sources of RKN resistance have been utilized in Upland cotton *G. hirsutum*, NemX (Ogallo et al., 1999; Wang et al., 2006a; Roberts and Ulloa, 2010), Clevewilt 6 and derived lines Stoneville LA887 and Paymaster H1560 (Robinson et al., 2001), and Auburn 623 and its derivatives (Shepherd, 1974; McPherson et al., 2004). Highly resistant Auburn 623 RNR was derived from two moderately resistant parental lines, Clevewilt 6-1 and Mexico Wild Jack Jones. The RKN resistance sources Auburn 623 RNR, Auburn 634 RNR, and their derived N-lines (Hyer and Jorgenson, 1984) were reported to be transgressive segregants (Shepherd, 1974; Hyer et al., 1979). Wang C. et al. (2008) reported that a segregating factor (*RKN2*) from susceptible parent *Gossypium barbadense* Pima S-7 could not function alone but interacted with a major recessive gene *rkn1* in *G. hirsutum* NemX (Wang et al., 2006b) to produce a higher resistance phenotype than resistant parental line NemX in progeny of the interspecific cross between Pima S-7 and NemX. Interestingly, the two genes *RKN2* and *rkn1* were mapped to the same region on Chr 11 (Wang C. et al., 2008). Transgressive resistance was also observed in some intraspecific F_2:7_ (NemX × SJ-2) RI (recombinant inbred) homozygous resistant lines, indicating susceptible parent SJ-2 contributed to higher resistance in progeny than the *rkn1* resistance contributed by NemX alone (Wang et al., 2006a). Genetic mapping and quantitative trait loci (QTL) analysis suggested a major telomeric segment on Chr 11 harbors RKN resistance genes from these different resistance sources (Bezawada et al., 2003; Shen et al., 2006, 2010; Wang et al., 2006b; Wang C. et al., 2008; Gutiérrez et al., 2010; Roberts and Ulloa, 2010; Ulloa et al., 2010). A microsatellite marker (SSR) CIR 316 tightly linked to resistance gene *rkn1* and other RKN resistance genes on Chr 11 was identified in different segregating populations (Shen et al., 2006, 2010; Wang and Roberts, 2006b; Wang et al., 2006b; Ynturi et al., 2006; Gutiérrez et al., 2010; Roberts and Ulloa, 2010; Ulloa et al., 2010). A resistance gene contributing to suppression of nematode egg production originally derived from Wild Mexico Jack Jones also was identified on Chr 14 (Gutiérrez et al., 2010; He et al., 2014; Kumar et al., 2016), which in combination with a resistance gene on Chr 11 derived from Clevewilt produced transgressive resistance in Auburn 623.

In a parallel study using an interspecific RIL population from a cross between two susceptible parents (*G. hirsutism* TM-1 × *G. barbadense* Pima 3-79), Wang C. et al. (2012) identified four major QTLs (on Chr 3, 4, 11, and 17) and two major QTLs (Chr 14 and 23) which contributed 8–12% transgressive resistance to nematode root-galling (galling index, GI) and nematode reproduction (eggs per gram root, EGR), respectively. In addition, 19 and 15 minor QTLs were identified in the TM-1 × Pima 3-79 population with each QTL accounting for 4–7% of phenotypic variance in GI and EGR, respectively (Wang C. et al., 2012). Although each of these QTLs contributed minor effects on phenotype, combinations of two to four major and/or minor QTLs were shown to dramatically reduce root-galling and nematode egg production by >50%, suggesting epistatic effects among these QTLs (Wang C. et al., 2012).

While these studies establish that transgressive segregation is common in cotton for disease resistance, the underlying functional mechanisms for this phenomenon are not known. For example, the location of the transgressive factor in SJ-2 in RIL population F_2:8_ (NemX × SJ-2) is not known, nor whether it would play a positive role on resistance in other recombinant populations. Other knowledge gaps include the nature of inheritance of the transgressive factor *RKN2* in *G. barbadense* Pima S-7 and how it behaves in later generations of the RIL F_2:7_ (Pima S-7 × NemX) population; and how genes interact in the region of *rkn1* in different genetic backgrounds. Previous studies demonstrated that a QTL mapping approach could be informative for studying inheritance and gene action to determine further the mechanism of transgressive segregation (Ulloa et al., 2010; Wang C. et al., 2012). Therefore, in this study, we report the development of a new RIL population (Pima S-7 × NemX) and its use in fine-mapping the *rkn1* region on Chr 11 and characterizing transgressive resistance with new molecular markers developed from the *G. raimondii* D_5_ genome reference sequence (Paterson et al., 2012). Through QTL analysis the transgressive segregation for RKN resistance was characterized further in six other genetic populations derived from three parental lines, *G. barbadense* Pima S-7, *G. hirsutum* NemX and SJ-2, and a simple genetic model for transgressive segregation in the Chr 11 region is proposed.

## Materials and methods

### Plant materials and crosses

Three highly inbred homogeneous parental lines, susceptible *G. hirsutum* cv. SJ-2 (USDA-ARS), resistant *G. hirsutum* cv. NemX (Bayer Corp., formerly by CPCSD; Oakley, 1995), and susceptible *G. barbadense* Pima S-7 (USDA-ARS) were utilized. Three F_1_ populations were developed from crosses between these genotypes. The F_2_ population was developed by self-pollinating the F_1_ hybrid between two parents. The RIL population was derived by single-seed descent from the F_2_ generation to develop highly homozygous generations. Interspecific crosses included 106 F_2_ (Pima S-7 × SJ-2) plants for nematode phenotyping of which 100 were used for mapping and QTL analysis, 108 RI Pima S-7 × NemX lines (90 F_2:7_, 8 F_2:6_, and 10 F_2:5_) for nematode phenotyping and QTL mapping, plus 165 F_2_ (Pima S-7 × NemX) plants and 51 plants of testcross NemX × F_1_ (Pima S-7 × SJ-2; Wang C. et al., 2008) for QTL mapping. Intraspecific crosses included 96 F_2_ (NemX × SJ-2) plants, 69 F_2:8_ (NemX × SJ-2) RIL families, 97 BC_1_F_1_ (NemX × F_1_) plants, and 48 BC_1_F_1_ (SJ-2 × F_1_) plants (Wang et al., 2006b).

### Nematode resistance screening

Nematode resistance phenotyping followed the method of Wang et al. (2006a,b). Nematode resistance in cotton populations was screened under controlled greenhouse conditions at 28–35°C during the day and 24°C at night. Five replicate plants of each line in the RIL population and of parental lines were arranged in a complete randomized design. One seed per plastic pot (10-cm-diam. × 17-cm-deep) was sown in blow sand and grown for 3 weeks. Then each plant was inoculated with ~50,000 eggs of *M. incognita* race 3 (isolate Project 77) and fertilized with slow-release fertilizer (Scotts-Sierra Horticultural Products Co) (Wang et al., 2006a). Egg extraction was conducted in NaOCl (Hussey and Barker, 1973). Infection response on cotton plants was evaluated at 60 days post-inoculation using the root-galling scale modified from Bridge and Page (1980), with 0 (no symptom) −10 (severe symptom). The number of nematode eggs per gram fresh root was also utilized for nematode infection response on cotton. Resistance threshold in each test was determined by the mean values of galling index and eggs per gram root of parents plus standard deviation (SD) (Wang et al., 2006a).

### Data analysis

Data analysis followed the methods of Wang C. et al. (2008). One-way ANOVA was used to analyze data. The treatment means were compared with Fisher's Protected LSD test. The transformed log_10_ (x + 1) data for nematode egg production were used for analysis. Chi-square (goodness of fit) test was used to predict Mendelian inheritance ratios.

### Marker analysis

Since chromosomes 11 and 14 are associated with root-galling index and/or nematode egg production (Shen et al., 2006; Wang et al., 2006b; Gutiérrez et al., 2010; He et al., 2014), markers on Chr11 and its homoeologous chromosome 21, and markers on Chr 14 were screened for nematode resistance linkage in the RIL population Pima S-7 × NemX. In addition, polymorphic markers linked to nematode resistance or Fusarium wilt resistance in the RIL population Pima 3-79 × TM1 (Ulloa et al., 2011, 2013; Wang C. et al., 2012; Wang et al., 2015; www.cottonmarker.org) were also used for screening RIL population Pima S-7 × NemX. A total of 186 SSR markers containing 366 polymorphic alleles across the whole genome were obtained for QTL analysis of nematode resistance.

DNA was extracted from fresh or frozen (−80°C) young cotton leaves using the DNeasy® Plant Mini kit (Qiagen, Valencia, CA, USA; Wang et al., 2006b). Primers were synthesized by IDT (IDT, Coralville, IA, USA). The forward primers were synthesized with an M13 forward sequence on the 5′-end (Wang et al., 2006b). The IRD labeled M13 primer (700 or 800 channel: CACGACGTTGTAAAACGAC) was made by LI-COR (LI-COR, Lincoln, NE, USA). The methods of PCR amplification of cotton molecular markers and of electrophoresis and detection were described by Ulloa et al. (2016). The SCAR marker GHACC1 and AFLP markers M9E2, M5E1, and M4E5 linked to *rkn1* were from Wang and Roberts (2006b).

### Fine-mapping region *rkn1* on Chr 11 and Chr 21 with D_5_ genome

In order to fine-map the resistance region of *rkn1*, 68 primers (Supplemental file 1: Table S1) were designed from about 2.2 M bp of sequence which extended from 58.2 to 60.4 M bp on chromosome 7 (corresponds to Chr 21 in tetraploid cotton) of the diploid D_5_ genome (*G. raimondii* v.1.1; Paterson et al., 2012; https://phytozome.jgi.doe.gov) containing markers MUCS088, CIR316 and CIR069 which are associated with RKN resistance (Wang et al., 2006b; Wang C. et al., 2008). The primers were named as UCR+ number, such as UCR1 or UCR2. The 2.2 M bp sequence contained markers CIR316 and CIR069 which were linked to *rkn1* or other resistance genes from different resistance sources on Chr 11 (Shen et al., 2006; Wang et al., 2006b). The parental lines Pima S-7, NemX and SJ-2 were screened for polymorphism with the 71 primers. The identified polymorphic markers were used to screen segregating populations F_2_ (NemX × SJ-2), F_2:8_ (NemX × SJ-2), RIL Pima S-7 × NemX, and test-cross NemX × F_1_ (Pima S-7 × SJ-2).

### Genetic linkage and QTL analysis

The linkage groups for chromosomes were developed by using the JoinMap^R^ version 4.0 program (Van Ooijen, 2006). The Kosambi map function was used to examine Logarithm of odds (LOD) scores of 3–15 for each population. To determine linkage between any two markers, a maximum distance of 40 centiMorgans (cM) and a LOD threshold score >4.0 were used. QTL analyses were conducted on galling index (GI) and egg production per gram root (LogEGR) using MapQTL 5.0 (Van Ooijen, 2004). Non-parametric mapping [Kruskal-Wallis analysis (K^*^)] test equivalent to the one-way analysis of variance was used for single-marker analysis and interval mapping for analysis of pairs of linked markers. Significant QTLs were set with a more stringent *P* < 0.005 for the K^*^-test (Wang C. et al., 2012).

## Results

### Phenotyping F_2:7_ (pima S-7 × NemX)

**Figure 1:**
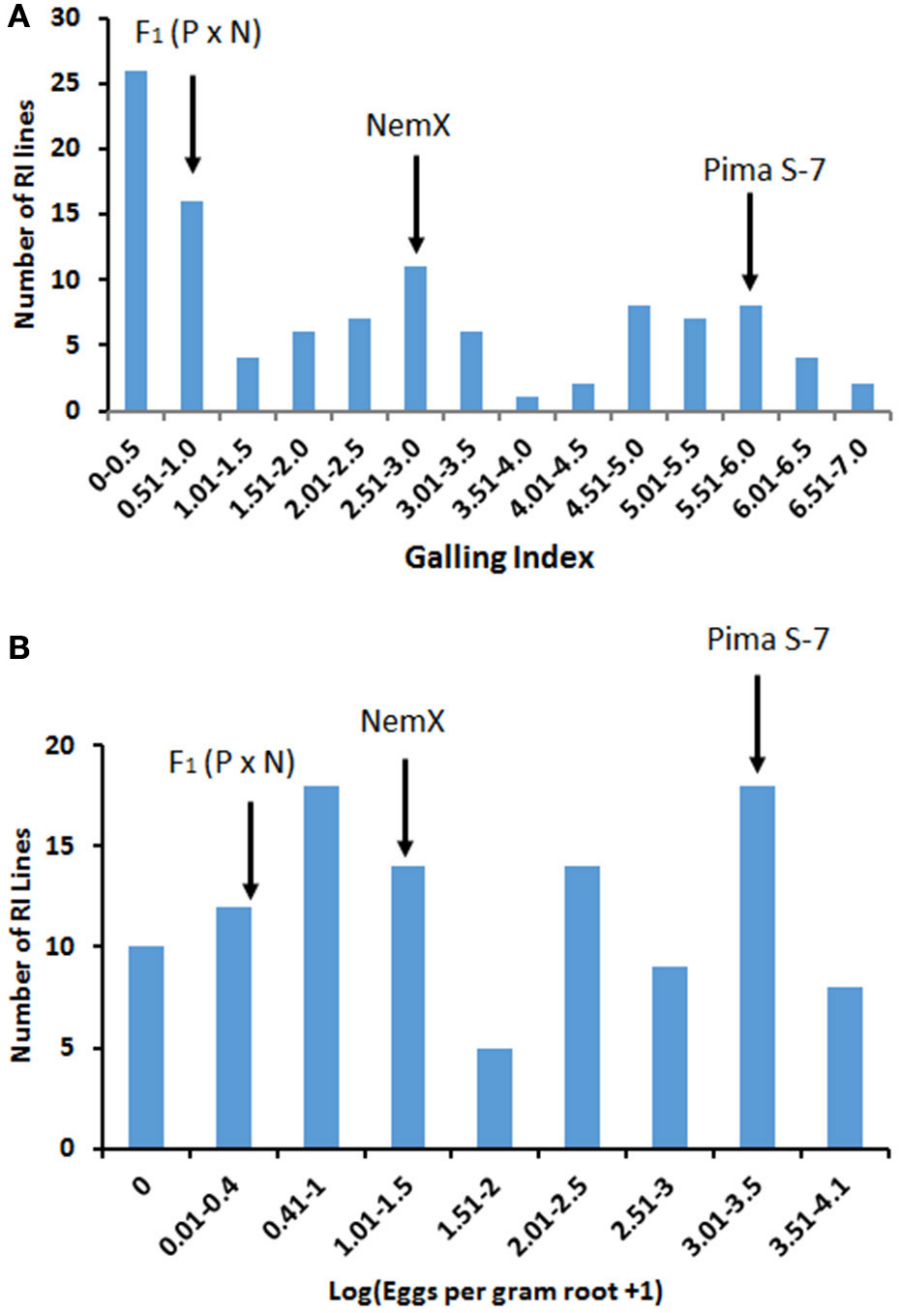
Distribution of *Meloidogyne incognita* root-galling (galling index, GI) in the Pima S-7 (P) × NemX (N) F_2:7_ RIL population **(A)**, and *M. incognita* egg production [Log(EGR+1)] in the Pima S-7 × NemX F_2:7_ RIL population **(B)**. The F_2:7_ population was evaluated at 60 days after inoculation. Galling index: 0–10 scale; 0 no galling, and 10 severe galling. Arrows point to the mean phenotypic reaction of the parents and F_1_ (P × N).

Fifty-six of 108 RI lines had lower (*P* < 0.05) GI than parent NemX (2.7 ± 0.27, SD) accounting for 51.9 % transgressive resistance (Figure 1A) and 39 lines had lower (*P* < 0.05) LogEGR (1.35 ± 0.81, SD) accounting for 36.1% transgressive resistance (Figure 1B). Seventy-one lines with GI < 3 accounted for 66% resistance in the RIL population. Ten of 108 lines had higher LogEGR than parent Pima S-7 (3.33 ± 0.16, SD) accounting for 9.3% transgressive susceptibility (Figure 1A). A high correlation (*R*^2^ = 0.70) was found between GI and LogEGR. These results confirmed that genes from both parents play a role in determining the resistance phenotype in these transgressive segregants from the F_2:7_ population.

### Fine mapping region *rkn1* in RIL populations F_2:7_ (pima S-7 × NemX) and F_2:8_ (nemx × SJ-2) with D_5_ genome sequence

**Figure 2:**
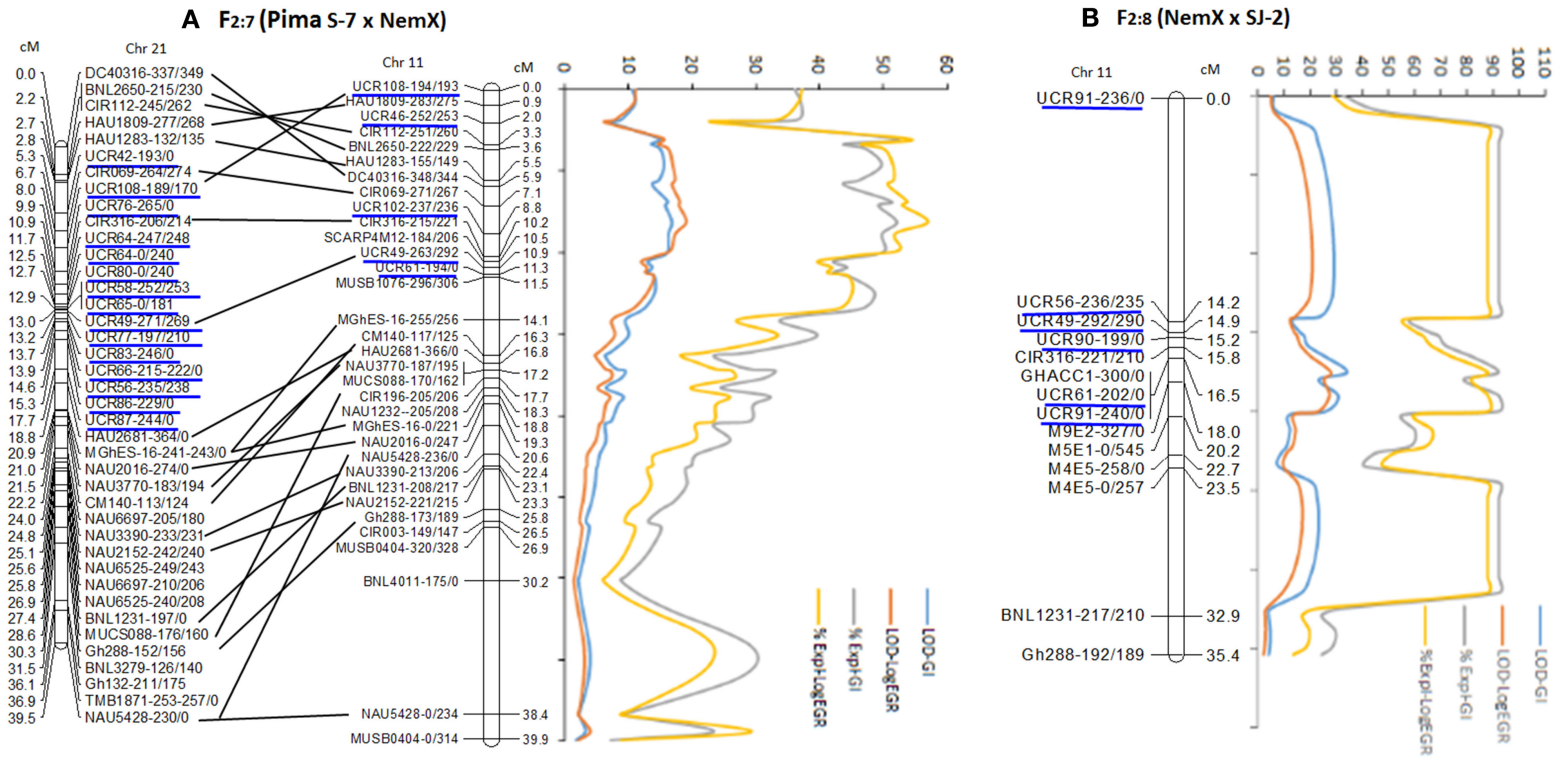
Fine mapping of *rkn1* region on chromosome (Chr) 11 and its homoeologous Chr 21 in RIL population F_2:7_ (Pima S-7 × NemX) **(A)** and *rkn1* region on Chr 11 in F_2:8_ (NemX × SJ-2) **(B)** using D_5_ genome sequence **[41]**. New markers are underlined in blue. Numbers after the marker name indicate the allele sizes after PCR amplification from the two parent lines, Pima S-7/NemX in the Pima S-7 × NemX RIL population and NemX/SJ-2 in the F_2:8_ (NemX × SJ-2) population. For example, for marker UCR108-194/193 in F_2:7_ (Pima S-7 × NemX), 194 bp amplified from Pima S-7 and 193 bp from NemX. All allelic sizes include the M13 primer tail.

Twenty of 68 SSR primers designed from D_5_ sequence produced 29 polymorphic markers between Pima S-7 and NemX and six polymorphic markers between NemX and SJ-2 [the primer sequences, their positions on *Gossypium raimondii* (v.2.1) (https://phytozome.jgi.doe.gov), *G. hirsutum* (pre-released v.1.1) (https://phytozome.jgi.doe.gov), and *G. arboreum* (https://blast.ncbi.nlm.nih.gov/Blast.cgi), and primer sequence function with JGI-Blast tool are provided in Supplemental file 1: Table S1]. Five polymorphic markers (UCR46, UCR49, UCR61, UCR102, and UCR108) were mapped to the *rkn1* region on Chr 11 over 11.3 cM and 15 mapped to the homoeologous region over 12.4 cM on Chr 21 in the F_2:7_ (Pima S-7 × NemX) (Figure 2A), with both regions containing a homozygous allele pair of SSR marker CIR316 associated with RKN resistance. Twenty-two of 32 markers on Chr 11 showed homozygous allele content on Chr 21 (Figure 2A). Five (UCR49, UCR56, UCR61, UCR90, and UCR91) of six polymorphic markers mapped to the region of CIR316 closely linked to *rkn1* expanded 2.3 cM in the F_2:8_ (NemX × SJ-2; Figure 2B). Of these, UCR90 and UCR91 produced 3 polymorphic markers between NemX and SJ-2, but not between Pima S-7 and NemX (Figure 2B).

### QTL mapping in F_2:7_ (pima S-7 × NemX) and F_2_ (pima S-7 × NemX) populations

#### F_2:7_ (pima S-7 × NemX)

A total of 395 (366 plus 29 listed above) polymorphic markers were used to genotype the F_2:7_ (Pima S-7 × NemX) for QTL mapping. Both Kruskal-Wallis analysis and interval mapping revealed that the significant (*P* < 0.0001) QTL(s) spanned 38 cM (0–38 cM) on Chr 11, associated with both GI and LogEGR phenotypes (Figure 2A, Supplemental file 2: Table S2A). The contribution to resistance to both galling and EGR was >40% in a 8.1 cM region spanning from 3.3 cM (CIR112-251/260) to 11.5 cM (MUSB1076-296/306) containing marker CIR316 linked to *rkn1* (Figure 2A). The region between the two flanked SSR markers CIR069-271/269 and UCR102-237/236 contributed 52% phenotypic variance of resistance to galling and 57% to egg production (Figure 2A), indicating the *rkn1* region contributed to both GI and LogEGR. Interval mapping demonstrated strong additive effects for resistance to both galling (1.62 for UCR102-237/236) and egg production (1.08 for CIR069-271/269; Supplemental file 2: Table S2A), suggesting an epistatic effect on phenotype. Resistance contribution of the locus CIR316-215/221 to both GI and LogEGR is shown in Table 1. Two other significant (*P* < 0.005) QTLs from Pima S-7 alleles, on Chr 3 (BNL3792-540/0) to GI, Chr 3 (MUSS396-0/300 to LogEGR, and Chr 1 (NAU4045-185/195) to both GI and LogEGR, accounted for ~7–10% phenotypic variance (Supplemental file 2: Table S2A). The homoeologous region on Chr 21 had no resistance contribution to GI or LogEGR even though the order of the marker alleles was similar to those on Chr11, and also no contribution to resistance was found on Chr 14.

**Table 1:** Resistance contribution of CIR316 locus in seven interspecific and intraspecific populations.

**Populations**	**Locus**	**Root galling (GI)**					**Nematode reproduction (LogEGR)**				
		**Kruskal-Wallis Analysis**		**Interval Mapping**			**Kruskal-Wallis Analysis**		**Interval Mapping**		
		K^*^	**Signif**.	**LOD**	**% expl**	**additive**	K^*^	**Signif**.	**LOD**	**% expl**	**Additive**
**INTERSPECIFIC CROSS**											
F_2_ (Px N)	CIR316-215/221	26.7	0.0001	6.5	17	0.91513	33.0	0.0001	7.4	19.3	0.47932
F_2:7_ (PXN)	CIR316-215/221	44.3	0.0001	15.7	48.2	1.56367	48.8	0.0001	14.9	46.3	1.00325
F_2_ (PXS)	CIR316-215/210	15.7	0.0001	3.5	15	−1.05808	22.8	0.0001	5.8	23.7	−0.32939
NemX × F_1_ (PXS)	CIR316-215/210	25.9	0.0001	9.4	57.3	−1.41808	25.6	0.0001	6.9	46.5	−0.66992
**INTRASPECIFIC CROSS**											
F_2_ (NXS)	CIR316-221/210	33.7	0.0001	9.2	35.8	−0.95977	26.5	0.0001	5.8	24.1	−0.53658
F_2:8_ (NXS)	CIR316-221/210	41.3	0.0001	19.1	72	−2.22059	40.9	0.0001	16.3	66.4	−0.95409
BC_1_F_1_ NemX × F_1_ (NXS)	CIR316-221/210	39.2	0.0001	12.1	43.8	−1.87151					
BC_1_F_1_ SJ-2 × F_1_ (NXS)	CIR316-210/221	23.0	0.0001	6.0	44.2	−1.44089					

*P, Pima S-7, allele 215 bp; N, NemX, allele 221 bp; S, SJ-2, allele 210 bp*.

*%expl, percentage of phenotypic variance explained*.

K^*^, Kruskal-Wallis analysis test regraded as the nonparametric equivalent of the one-way analysis of variance.

*Signif., significance level*.

#### F_2_ (pima S-7 × NemX)

In order to confirm the *rkn1* region on Chr11, markers CIR316, CIR069, and NAU2016 were used to screen 165 F_2_ (Pima S-7 × NemX) plants which were phenotyped in our previous study (Wang C. et al., 2008). QTL mapping confirmed that the three markers on chr11 were associated with resistance. CIR069 contributed 18.5% phenotypic variance to GI and 23% to LogEGR with additive effects (GI: 0.91454; LogEGR: 0.47609) based on interval mapping.

### QTL mapping in testcross NemX × F_1_ (pima S-7 × SJ-2)

**Figure 3:**
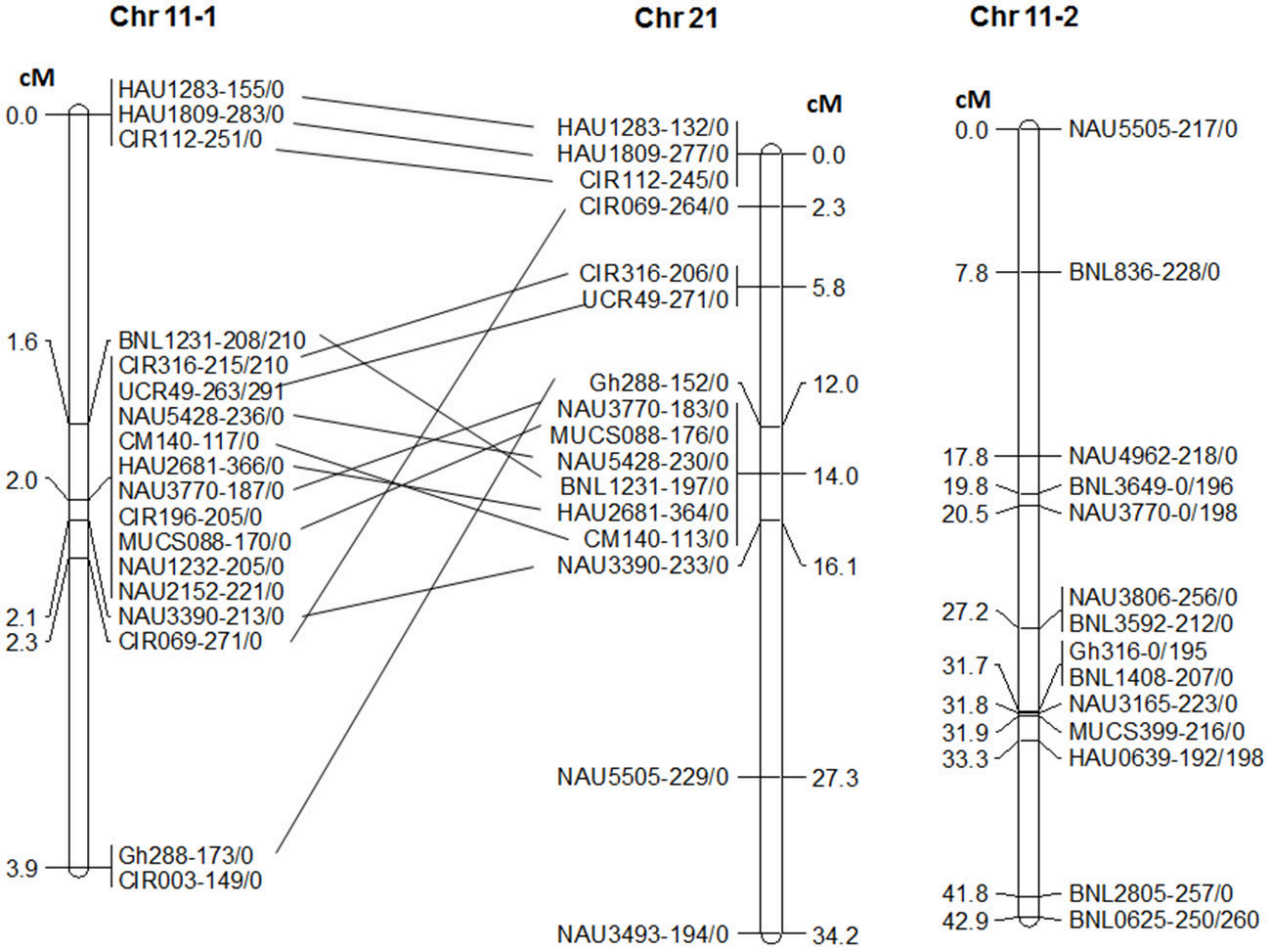
Linkage groups of chromosome (Chr) 11 and homoeologous Chr 21 in testcross population NemX × F_1_ (Pima S-7 × SJ-2). Chr 11-1 represents the *rkn1* region at the telomeric end of Chr 11 and Chr 11-2 represents the lower part of this chromosome close to the centromere. Numbers after the marker name indicate the allele sizes after PCR amplification from the two lines Pima S-7/SJ-2. All allelic sizes include the M13 primer tail.

In order to confirm the location of *RKN2* in the same region of *rkn1* [17], 32 SSR primers on Chr 11 were screened with the testcross population NemX × F_1_ (Pima S-7 × SJ-2) and produced 53 polymorphic markers between Pima S-7 and SJ-2. Of these, 32 were mapped on Chr 11 and 16 on Chr 21 (Figure 3). Interestingly, 18 markers including CIR316 and CIR069 on Chr 11-1 were clustered together in a 3.9-cM interval but over a 16.1 cM interval on the homoeologous region of Chr 21 (Figure 3). Marker MUCS088 closely linked to *RKN2* was mapped to the same position as CIR316. Fourteen other markers without clustering were mapped to a region further from *rkn1* spanning 42.9 cM (Figure 3, Chr 11-2).

QTL analysis confirmed that the mapped *rkn1* region (3.9 cM) was significantly (*P* < 0.0001) involved in both GI and LogEGR phenotypes in the testcross population (Supplemental file 2: Table S2B). Interval mapping analysis revealed that the CIR069 region on Chr 11 contributed up to 62.5% phenotypic variance in GI and 50.3% in LogEGR. Again, there was no contribution to resistance phenotype from Chr 21 and Chr 14.

**Figure 4:**
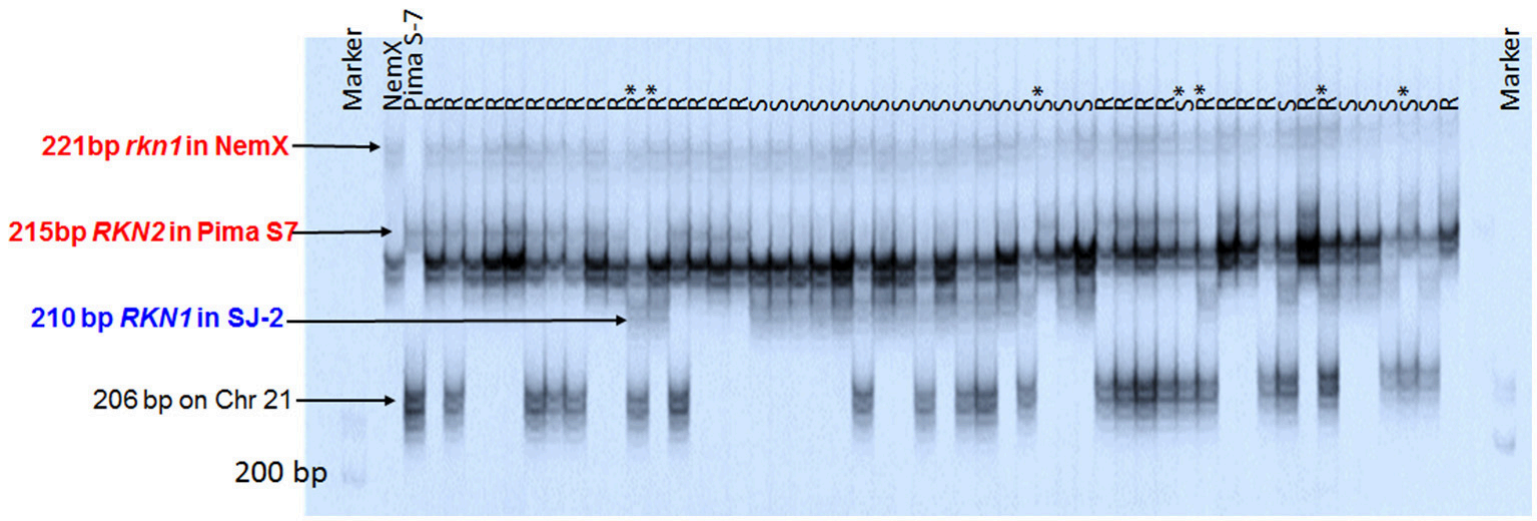
Image of amplification products with SSR marker CIR316 in the segregating population NemX × F_1_ (Pima S-7 × SJ-2) on polyacrylamide gel with model 4000 LI-COR IR2 automated sequencer. R, resistant; S, susceptible, all based on phenotype. R^*^ and S^*^, recombinant line with resistant phenotype (R) and susceptible marker profile or susceptible phenotype (S) and resistant marker profile, respectively. Marker: 50–350 bp sizing standard. Arrows point to the marker allele positions. All allelic sizes include the M13 primer tail.

Among the 18 markers clustered around *rkn1*, only CIR316 and BNL1231 had polymorphism among three parental lines (Pima S-7, NemX, and SJ-2). The application pattern of CIR316 clearly showed that all lines carried the heterozygous allele (221 bp) linked to *rkn1* from NemX (Figure 4). The Pima S-7 allele band (215 bp) was present in the resistant lines and the SJ-2 allele band (210 bp) was present in the susceptible lines except for seven out of 51 lines for which recombination had occurred between CIR316 and the resistance gene based on galling index (Figure 4). An additional Pima S-7 allele band (206 bp) was mapped on Chr 21 (Figure 4).

### Phenotyping and QTL mapping an F_2_ (pima S-7 × SJ-2) population derived from susceptible parents

**Figure 5:**
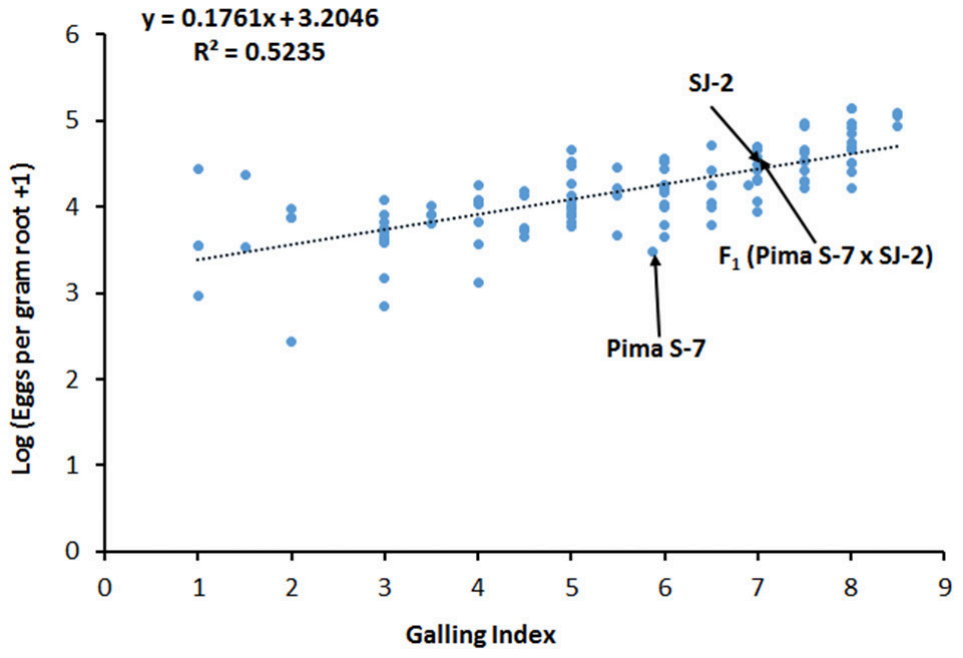
The relationship between galling index and egg production in the F_2_ (Pima S-7 × SJ-2) population 60 days after inoculation. Log_10_(x+1) transformed data were used for analysis of eggs per gram of root. Galling index: 0–10 scale; 0 no galling, and 10 severe galling. Mean data points for the Pima S-7 and SJ-2 parents and their F_1_ are indicated.

Since the interactions in the *rkn1* region are complex, we examined whether transgressive segregation occurred in the F_2_ (Pima S-7 × SJ-2) population derived from two susceptible parents and determined whether transgressive factor *RKN2* functions in the F_2_ (Pima S-7 × SJ-2) without the *rkn1* allele. The correlation (*R*^2^) between galling index and egg production per gram root was 0.5235 (Figure 5). The phenotypic test revealed that 52 out of 106 (49%) F_2_ (Pima S-7 × SJ-2) plants showed less GI than Pima S-7 (5.85 ± 0.22, SD), SJ-2 (7 ± 0.61, SD), and their F_1_ (Pima S-7 × SJ-2; 6.9 ± 0.42, SD). Twenty out of 106 F_2_ plants had low GI < 3 (range 1–3) accounting for 19% resistance. The results indicated that both susceptible parents contributed to transgressive resistance. QTL mapping indicated that the CIR 316-215 bp allele from Pima S-7 on Chr 11 contributed 15% phenotypic variance to GI and 24% to LogEGR, respectively (Table 1, Supplemental file 2: Table S2C).

### QTL mapping of intraspecific cross NemX × SJ-2

#### F_2:8_ (NemX × SJ-2)

A set of 62 genome-wide markers polymorphic between NemX and SJ-2, including one SCAR marker (GHACC1) and four AFLP markers [37] associated with the *rkn1* locus in NemX were screened for QTL analysis. QTL mapping showed that markers UCR91, UCR61, and GHACC1 tightly linked to the CIR316-221/210 marker contributed 82.1% phenotypic variance for GI and 81.7% for LogEGR around the region of *rkn1* (Figure 2B, Table 1, Supplemental file 2: Table S2D), confirming that this same *rkn1* region played a similar role in resistance to both GI and LogEGR. No resistance contribution was found from Chr 21 and Chr 14.

#### F_2_ (NemX × SJ-2)

QTL mapping using 40 polymorphic markers indicated that the CIR316 region on Chr 11 had a major effect on both galling and EGR (Supplemental file 2: Table S2E), which confirmed a previous report (Wang et al., 2006b). The CIR 316-221/210 marker contributed 36% phenotypic variance in galling and 24% in LogEGR (Table 1), and the AFLP-derived SCAR marker GHACC1 [37] which was 1.6 cM from CIR316 showed 36% phenotypic variance in galling and 33% in LogEGR (Supplemental file 2: Table S2E), emphasizing that the same *rkn1* region contributed to both GI and EGR. No resistance contribution was found from Chr 14 and Chr 21.

#### BC_1_F_1_ NemX × F_1_ (NemX × SJ-2) and BC_1_F_1_ SJ-2 × F_1_ (NemX × SJ-2)

The CIR316-221/210 marker in BC_1_F_1_ population NemX × F_1_ (NemX × SJ-2) closely linked to *rkn1* contributed 43.8% to phenotypic variance in galling (Table 1). The CIR316-215/210 marker in the BC_1_F_1_ population SJ-2 × F_1_ (NemX × SJ-2) in which lower galling index was found in progenies than both susceptible parental lines contributed 44.2% phenotypic variance in GI (Table 1), also indicating the *rkn1* region from both NemX and SJ-2 plays an important role for nematode transgressive resistance in the intraspecific cross.

The increase in resistance by SJ-2 in progenies from both the Pima S-7 crosses with SJ-2 and the NemX crosses with SJ-2 is revealed by the CIR316 210 bp allele from SJ-2 and confirms that the transgressive factor in SJ-2 (designated as *RKN3*) is linked to *rkn1* from NemX and to *RKN2* from Pima S-7.

## Discussion

Analysis of RKN resistance phenotypes in multiple segregating cotton populations in this study established the significant and common occurrence of transgressive nematode resistance among progenies of both interspecific and intraspecific populations. Transgressive segregation occurred in both interspecific crosses between *G. barbadense* Pima S-7 and *G. hirsutum* NemX, and between Pima S-7 and *G. hirsutum* SJ-2, and also in the *G. hirsutum* intraspecific cross between NemX and SJ-2. The results also establish that in the case of the crosses with resistant NemX which carries the recessive R gene *rkn1* (Wang et al., 2006b), the susceptible parent in each case, whether intraspecific (SJ-2) or interspecific (Pima S-7) contributes at least one transgressive factor that enhances resistance. More unexpectedly, the interspecific cross between two susceptible parents [Pima S-7 (*RKN2*) (Wang C. et al., 2008) and SJ-2 (designated as *RKN3*)] also results in combinations of transgressive factors which produce some progenies with resistance phenotypes. Beside the R gene *rkn1*, these resistance-enhancer factors may be the results of epistasis or complex recombinations and interactions of tandemly arrayed alleles (TAA) or genes (TAG). Copy number of multiple genes in a 31-kb segment at the *Rhg1* locus for resistance to cyst nematode in soybean has been shown to determine the level of expressed resistance, with copy number ranging from 1 (susceptible), to 3 (partially resistant) to 10 (highly resistant) in different genotypes (Cook et al., 2012). Whether or not a comparable arrangement of multiple genes with varying copy number determining the level of resistance expression occurs in the cotton *rkn1* region will require identification of the gene sequences determining resistance phenotype.

The refined mapping by QTL analysis of the genome region harboring *rkn1* on Chr 11 and its homoeologous region on Chr 21 provided further insight into the transgressive resistance control by this region. Of the original 40 cM mapped region of *rkn1* which contributed resistance to both root-galling and egg production phenotypes in the F_2:7_ (Pima S-7 × NemX) population, 8 cM of the region containing marker CIR316 accounted for more than 40% of the phenotypic variance. This result indicated that genes cluster together in the region but cannot be separated only by phenotype. Strong positive additive effects on resistance phenotypes were found in both the F_2_ and F_2:7_ (Pima S-7 × NemX) RIL populations, suggesting that epistasis plays a role in the RKN transgressive resistance in this interspecific cross. Although the interval bracketed by markers HAU1809 and Gh288 in the *rkn1* region was 25–27 cM in length on Chr 11 and Chr 21, in the testcross population we found at least three-fold shorter interval on Chr 11 than on Chr 21, indicating complex recombination between the *rkn1* region and its homoeologous Chr 21. This shorter genetic distance on Chr 11 in the testcross population, which contributed 50–60% phenotypic variance, suggested that the complex recombination in the *rkn1* region produced transgressive resistance and that the level of resistance depends on the specific parent combination (Ulloa et al., 2010). The non-separated markers might result from homologous/repetitive sequence or repetitive transposable elements in the *rkn1* region (Wang et al., 2015). In addition, more QTLs around the *rkn1* region in RIL population NemX × SJ-2 (Figure 2B) might exist but were not detected because of fewer polymorphic markers available between NemX and SJ-2, suggesting more complexity in this region.

The homoeologous region of chromosome 21 consistently failed to show any detectable resistance contribution even though the order of marker alleles amplified from the same primer pair appeared similar in various segregating populations developed from crosses between the three parental lines. Although the sequence is highly conserved between Chr 11 and Chr 21 (Paterson et al., 2012; Wang K. et al., 2012; Li et al., 2014, 2015; Zhang et al., 2015), only Chr 11 contributed to RKN resistance, emphasizing that the unique structure and gene combination on Chr 11 is the primary basis for RKN resistance control and that minor differences between Chr 11 and Chr 21 result in phenotypic change, as reported earlier with only one nucleotide difference (SCAR marker GHACC1) between NemX and SJ-2 causing phenotypic change for RKN resistance (Wang and Roberts, 2006b). An additional result of importance from this study was that no contribution of resistance to root-galling or egg production was found on Chr 14, supporting that the resistance in NemX is based on *rkn1*, and thus different from the resistance in other cotton lines such as Wild Mexico Jack Jones, Auburn 623 and its derived M-lines which all carry a resistance gene on Chr 14 effecting egg production but not galling response (Gutiérrez et al., 2010; He et al., 2014; Kumar et al., 2016).

Analysis of the CIR316-215/210 marker alleles in the F_2_ (Pima S-7 × SJ-2) population, which accounted for 15–24% of phenotypic variance, confirmed that the 215 bp allele in Pima S-7 enhanced resistance to both root-galling and egg production. We identified F_2_ progenies with GI phenotypes outside the range of the susceptible parents Pima S-7 and SJ-2, including ones with high resistance (GI < 3) and others with about 50% higher GI than the susceptible parents. Thus, both transgressive resistant and transgressive susceptible progenies were present in the F_2_, providing evidence that both parents contributed to the transgressive resistance. In this F_2_ population, which lacks the *rkn1* locus resistance, the transgressive factor in Pima S-7 (*RKN2*) might be dominant over the transgressive factor(s) in SJ-2 (*RKN3*) and allele interaction might occur in the region of CIR316, as found in the testcross. The behavior of *RKN2* from Pima S-7 showed dominant action in our previous study (Wang C. et al., 2008), where plants heterozygous or homozygous for *RKN2* were effective in enhancing resistance phenotype in the presence of *rkn1*. Comparing different generations of Pima S-7 × NemX progenies, about 25% transgressive resistant lines were found in the F_2_ population and in F_2:3_ families (Wang C. et al., 2008) and 52% in the F_2:7_ RIL population in this study. It is likely that this is also the situation in progenies with *RKN2* coupled with *RKN3* in Pima S-7 × SJ-2 crosses, but to result in transgressive resistant phenotypes, the SJ-2 factor (*RKN3*) must be in the homozygous condition. This would explain transgressive progenies occurring in the F_2_ but not in the F_1_ which is susceptible to RKN (Wang et al., 2006b).

Further aspects of the transgressive behavior contributed by the susceptible SJ-2 factor *RKN3* were revealed in the NemX × SJ-2 RIL population, in which SJ-2 markers closely linked to CIR316 contributed up to 82% of resistance to root-galling. This result indicated that the *RKN3* factor from SJ-2 also interacts with *rkn1*-based resistance in NemX in the *rkn1* resistance region. In our previous study of this population, about 62% of the RILs carrying the homozygous *rkn1* resistance were significantly more resistant than the level of resistance in the *rkn1* donor parent NemX and up to 30% of total RI lines showed transgressive resistance (Wang et al., 2006a,b). It is likely that this highly resistant set of RILs, which we confirmed to be homozygous for the recessive *rkn1* from NemX, were also homozygous for the *RKN3* transgressive resistance factor contributed by SJ-2. This was also confirmed in the BC_1_F_1_ population SJ-2 × F_1_ (NemX × SJ-2) in which the marker CIR316-210/221 contributed up to 44% of phenotypic variance to GI resistance. In the BC_1_F_1_, transgressive progenies showed less galling than both susceptible parent lines [SJ-2 and F_1_ (NemX × SJ-2)]. The stronger transgressive resistance which occurred in later than in early generations in the intraspecific NemX × SJ-2 cross compared with interspecific crosses between Pima S-7 and NemX or SJ-2 may reflect the increased levels of homozygosity of the interacting factors. Further, the presence of either homozygous or heterozygous *rkn1* improves resistance, and the increasing contribution of resistance is influenced by different recombination backgrounds.

Based on GI and LogEGR phenotypic data in the *rkn1* region in different populations and QTL analysis, we constructed a simple genetic model to better conceptualize the transgressive resistance among the three parental lines and their progenies (Table 2). The inferred genetic model is based on the linkage of the pairs of loci (*rkn1* with *RKN2, rkn1* with *RKN3*, and *RKN2* with *RKN3*) as indicated by mapping the respective alleles of the closely linked CIR316 marker.

**Table 2:** The three gene model in five segregating populations based on phenotypic data and QTL analysis.

	**NemX–N (*rkn1*) *aabbcc***	**Pima S-7–P (*RKN2*) *AABBcc***	**SJ-2–S (*RKN3*) *AAbbCC***
NemX – N (*rkn1*) *aabbcc*	*aabbcc* (R^**^)	*AaBb* (F_1PXN_, R) *AaCc* (F_1NXS_, S) Testcross N x F_1_ (PXS): Exp^#^:1R:1S (26R:26S) Obs: 29R:23S *χ^2^* = 0.692, *P* = 0.405 (Wang C. et al., 2008) GI^*^: 63.2%	*AaCc* (F_1_, S^**^) *aaC_*,*aacc* (R) F_2_ (NXSJ): Exp:1R:3S (24R:72S) Obs: 20R:76S *χ^2^* = 0.889, *P* = 0.346 (Wang et al., 2006b) GI: 37% F_2:8_ (NXSJ): Exp:1R:1S (34.5R:34.5S) Obs: 34R:36S *χ^2^* = 0.014, *P* = 0.904 (Wang et al., 2006a) GI: 92%
Pima S-7–P (*RKN2*) *AABBcc*	*aaB_*, AaB_, aabb (R) F_1_ (PXN): *AaBb* (R) F_2_(PXN): Exp:10R:6S (106R:63S) Obs: 105R:64S *χ^2^* = 0.025, *P* = 0.87 (Wang C. et al., 2008) GI: 30% F_7_(PXN): Exp: 1R:1S (54R:54S) Obs: 71R:37S *χ^2^* = 10.704, *P* = 0.001 GI: 51%	*AABBcc* (S)	*BBC_* (R); F_1_(PXSJ): *BbCc* (S) F_2_(PXSJ): Exp:3R:13S Obs: 20R:86S *χ^2^* = 0, *P* = 1 GI: 25%
SJ-2 – SJ (*RKN3*) *AAbbCC*			*AAbbCC* (S)

# *Chi-square test is based on galling index (GI) data since root galling and nematode reproduction had similar result*.

* *The number is based on the highest value of GI phenotypic variance in the interval mapping on the Chromosome 11 region*.

** *R, resistant phenotype; S, susceptible phenotype*.

Allele interactions and/or linked gene interactions in the *rkn1* region clearly play a major role in controlling transgressive resistance. We labeled the NemX resistance gene *rkn1* as *aa*, Pima S-7 transgressive factor *RKN2* as *BB*, and the SJ-2 transgressive factor *RKN3* as *CC*. Analysis of the F_2_ population (Pima S-7 × NemX) published previously (Wang C. et al., 2008), showed that the segregation for root-galling response followed a two-gene model for resistance (R, genotypes *AaB-, aaB-*, and *aabb*) and susceptibility (S, genotypes *AAB-* and *A-bb*). However, 66% resistant lines (71R:37S) based on GI were observed in the new F_2:7_ (Pima S-7 × NemX) RIL population which did not match the expected 1:1 ratio if only *rkn1* was involved. Since the transgressive resistance depended on the recombination background as discussed above, the greater proportion of resistant lines in this RIL population indicated that multiple genes or alleles are present in the *rkn1* region, which was also supported by significant effect QTL(s) spanning 38 cM (0–38 cM) in the *rkn1* region associated with both GI and LogEGR phenotypes. Extending the model, the exact fit to a 3R:13S ratio between resistant genotypes (*BBC-*) and susceptible genotypes (*BBcc, BbC-, bbC-*, and *bbcc*) in the F_2_ (Pima S-7 × SJ-2) population was predicted because both parents (*BBcc* and *bbcc*) and the F_1_ (*BbCc*) had susceptible phenotypes. The *RKN3* gene in SJ-2 (*AACC*), as with *RKN2* in Pima S-7, was expected not to express resistance on its own but produce transgressive resistance in the presence of homozygous *rkn1* because the F_1_ (NemX × SJ-2) (*AaCc*) was susceptible. This result might explain why in our earlier study (Wang et al., 2006a), 21 (*aaC-*) out of 34 resistant lines had higher resistance than the resistant parent NemX (*aacc*) in the NemX × SJ-2 RIL population for which a 3:1 ratio (25.5HR: 8.5R) for GI (χ^2^ = 3.176, *P* = 0.075) was found between the highly resistant (HR) genotype (*aaC-*) and resistant NemX type (*aacc*).

Considering the resistance contribution of the *rkn1* region in all seven segregating populations, the three resistance genes might represent multiple genes in tandem linkage, TAG or multiple alleles, TAA of unknown arrangement in one gene, possibly similar to the arrangement of resistance locus *Rhg1* in soybean (Cook et al., 2012). Although we did not have a population which allowed analysis of the three genes together, based on the interaction of the three possible gene pairs which we have analyzed here, the three possible combination pairs of the different loci or genes produce novel resistance (*aaB-, A-B-, aaC-* and *BBCC*) or susceptibility (*AAbb, AAcc, bbcc*) phenotypes in the progenies. The interspecific crosses (*aa* + *BB, BB* + *CC*) produced transgressive resistance (*aaB-, A-B-, aaC-* and *BBCC*) in earlier progeny generations than intraspecific crosses (*aa* + *CC*). In the presence of *rkn1* (*a-B- or aaCC*), stronger transgressive resistance occurred than without the *rkn1* gene (*AABB* and *BBCC*).

Sequence tandem repeats result from unequal crossing over during genetic recombination, and these tandem repeats (TR), TAAs, or gene cluster-TAGs are abundant across all domains of life. Little is known about their distribution and contribution to proteins. However, it is known that TR enriched leucine-rich repeats (LRRs) are commonly found in R genes (Schaper and Anisimova, 2015). Evidence of complex recombination between the *rkn1* region and its homoeologous chromosome 21 is presented in this study. The behavior of *RKN2* and *RKN3* showed the presence of TAAs or TAGs enhancing resistance phenotype in the presence of *rkn1*, resulting in transgressive resistant phenotypes. In addition, resistance to other soil-borne diseases mapped to Chr 11 also indicated the unique structure and resistance gene clustering on Chr 11 (Wang et al., 2015), such as found in resistance to reniform nematode (Dighe et al., 2009), Fusarium wilt (Ulloa et al., 2011, 2013), Verticillium wilt (Bolek et al., 2005), and black root rot (Niu et al., 2008). More research is needed to determine if TRs (e.g., SSRs), TAAs, or TAGs play a major role in transgressive resistance and facilitate resistance to emerging pathogen diseases.

Recently published genome-wide SNP or SSR linkage maps for fiber, yield, or other trait analysis (Yu et al., 2011, 2012; Zhao et al., 2012; Hulse-Kemp et al., 2015; Li et al., 2016; Iqbal and Rahman, 2017; Khan et al., 2017) and the available marker or genome sequence information might shed more light on the mechanism of transgressive resistance. For example, segregation distortion was commonly observed in cotton and greater genetic distance between parents could result in higher distortion (Yu et al., 2011; Zhao et al., 2012; Khan et al., 2017). The distortion could result from translocations, chromosome rearrangements, and other genomic structure variations (Khan et al., 2017). Therefore, deeper sequencing of Chr 11 would increase understanding of resistance gene clusters on Chr 11.

From a practical plant breeding standpoint, the SSR marker CIR316 not only has proven to be a powerful marker to study transgressive resistance in cotton, but also this marker plus the newly developed markers in the *rkn1* region reported here are important for marker-based breeding selection to develop nematode resistant cotton varieties. Moreover, through selection of highly resistant progenies resulting from transgressive segregation, valuable novel sources of resistance are available which can be tracked for breeding advancement by the reported marker profiles in the *rkn1* region. Although not proven, it is likely that the novel transgressive resistant progenies will be more durable than single source resistance when utilized in cotton production systems, because nematode populations frequently exposed to the resistance would have to overcome the combined effects of multiple allele or multiple gene action.

## Conclusions

In this study, QTL mapping of seven intraspecific and interspecific segregating populations generated from Pima S-7, NemX, and SJ-2 crosses revealed the allele profile of marker CIR316 in all tested populations corresponding with phenotypic profile, demonstrating that a transgressive factor (designated as *RKN3*) from susceptible SJ-2 exists in the region of *rkn1*. Allele or gene interactions between NemX (*rkn1*), SJ-2 (*RKN3*), and Pima S-7 (*RKN2*) contributed to transgressive resistance. Stronger transgressive resistance occurred in later than in early generations in the intraspecific cross NemX × SJ-2 but not in the interspecific cross. All positive contributions to resistance phenotype came from the *rkn1* region on Chr 11, while no evidence was found for any resistance contribution from the homoeologous region of Chr 21. The *rkn1* region had similar resistance contribution to both root-galling and egg production in each population. The complex TAA and/or TAG recombination and interactions in the *rkn1* resistance region in the NemX background provide a model to study transgressive resistance in plants.

## Author contributions

CW, PR, and MU conceived and designed the study. CW and TD performed the laboratory work. CW, PR, and MU analyzed the data and wrote the manuscript. All the authors read and approved the final manuscript.

### Conflict of interest statement

The authors declare that the research was conducted in the absence of any commercial or financial relationships that could be construed as a potential conflict of interest.

## Abbreviations

Chr: Chromosome
EGR: Eggs per gram root
GI: Galling Index
IM: Interval Mapping
K^*^ test: Kruskal-Wallis Analysis
RIL: Recombinant Inbred Line
RKN: Root-knot nematodes
QTL: Quantitative Trait Locus.
