# Supplementary material for: QTL Analysis of Transgressive Nematode Resistance in Tetraploid Cotton Reveals Complex Interactions in Chromosome 11 Regions
Source: Front Plant Sci. 2017 Nov 20;8:1979. doi: 10.3389/fpls.2017.01979 (PMC5702019; doi:10.3389/fpls.2017.01979)
Supplement: Supplementary file 2 [file Table2.docx]

**Supplemental file 2: Table S2.** QTL mapping of root galling (GI) and nematode egg production (LogEGR) in the interspecific and intraspecific populations.

| 1. **F_2:7_ (Pima S-7 x NemX)** | | | | | | | | | | | | | | | | | | | | | | | | | | | | | | | | | | | | | |
| --- | --- | --- | --- | --- | --- | --- | --- | --- | --- | --- | --- | --- | --- | --- | --- | --- | --- | --- | --- | --- | --- | --- | --- | --- | --- | --- | --- | --- | --- | --- | --- | --- | --- | --- | --- | --- | --- |
|  | |  | | |  | | | | GI | | | | | | | | | | | | | | | LogEGR | | | | | | | | | | | | | |
|  | |  | | |  | | | | Kruskal-Wallis Analysis (K* test) | | | | | | | Interval Mapping  IM | | | | | | | | K* test | | | | | | IM | | | | | | | |
| Chr | | Position | | | Locus | | | | K* | | | | Signif. | | | LOD | | | % Expl. | | Additive | | | K* | | | Signif. | | | LOD | | | | | % Expl. | | Additive |
| 11A | | 0 | | | UCR108-194/193 | | | | 34.129 | | | | 0.0001 | | | 10.72 | | | 36.2 | | 1.39027 | | | 41.755 | | | 0.0001 | | | 11.14 | | | | | 37.3 | | 0.923757 |
| 11A | | 0.858 | | | HAU1809-283/275 | | | | 34.467 | | | | 0.0001 | | | 11.11 | | | 37.2 | | 1.36614 | | | 38.659 | | | 0.0001 | | | 10.87 | | | | | 36.6 | | 0.886721 |
| 11A | | 2.003 | | | UCR46-252/253 | | | | 25.777 | | | | 0.0001 | | | 7.26 | | | 26.2 | | 1.15608 | | | 28.647 | | | 0.0001 | | | 6.36 | | | | | 23.4 | | 0.714803 |
| 11A | | 3.335 | | | CIR112-251/260 | | | | 36.856 | | | | 0.0001 | | | 13.62 | | | 43.9 | | 1.49386 | | | 46.745 | | | 0.0001 | | | 14.7 | | | | | 46.6 | | 1.00457 |
| 11A | | 3.639 | | | BNL2650-222/229 | | | | 40.251 | | | | 0.0001 | | | 15.09 | | | 47.4 | | 1.5483 | | | 51.072 | | | 0.0001 | | | 16.37 | | | | | 50.2 | | 1.04034 |
| 11A | | 5.54 | | | HAU1283-155/149 | | | | 42.706 | | | | 0.0001 | | | 14.9 | | | 46.4 | | 1.52833 | | | 57.571 | | | 0.0001 | | | 17.4 | | | | | 51.7 | | 1.05647 |
| 11A | | 5.852 | | | DC40316-348/344 | | | | 40.46 | | | | 0.0001 | | | 13.82 | | | 43.9 | | 1.48496 | | | 56.834 | | | 0.0001 | | | 16.92 | | | | | 50.8 | | 1.04472 |
| 11A | | 7.118 | | | CIR069-271/267 | | | | 46.819 | | | | 0.0001 | | | 15.87 | | | 49.3 | | 1.58923 | | | 60.878 | | | 0.0001 | | | 18 | | | | | 53.1 | | 1.07953 |
| 11A | | 8.843 | | | UCR102-237/236 | | | | 47.844 | | | | 0.0001 | | | 16.36 | | | 49.9 | | 1.61643 | | | 59.762 | | | 0.0001 | | | 17.42 | | | | | 51.9 | | 1.07922 |
| 11A | | 10.157 | | | CIR316-215/221 | | | | 44.334 | | | | 0.0001 | | | 15.71 | | | 48.2 | | 1.56367 | | | 48.8 | | | 0.0001 | | | 14.85 | | | | | 46.3 | | 1.00325 |
| 11A | | 10.526 | | | SCARP4M12-184/206 | | | | 38.092 | | | | 0.0001 | | | 13.11 | | | 42.2 | | 1.46226 | | | 42.008 | | | 0.0001 | | | 12.1 | | | | | 39.8 | | 0.928971 |
| 11A | | 10.908 | | | UCR49-263/292 | | | | 42.991 | | | | 0.0001 | | | 13.84 | | | 44.4 | | 1.50503 | | | 47.467 | | | 0.0001 | | | 12.97 | | | | | 42 | | 0.957318 |
| 11A | | 11.252 | | | UCR61-194/0 | | | | 41.244 | | | | 0.0001 | | | 13.06 | | | 42.1 | | 1.46203 | | | 47.863 | | | 0.0001 | | | 12.74 | | | | | 41.3 | | 0.947976 |
| 11A | | 11.456 | | | MUSB1076-296/306 | | | | 42.842 | | | | 0.0001 | | | 14.13 | | | 44.7 | | 1.50879 | | | 49.821 | | | 0.0001 | | | 14.07 | | | | | 44.6 | | 0.986372 |
| 11A | | 14.12 | | | MGhES-16-255/256 | | | | 33.784 | | | | 0.0001 | | | 9.63 | | | 33.9 | | 1.30607 | | | 29.845 | | | 0.0001 | | | 7.5 | | | | | 27.2 | | 0.766541 |
| 11A | | 16.296 | | | CM140-117/125 | | | | 23.15 | | | | 0.0001 | | | 6.41 | | | 23.5 | | 1.08832 | | | 18.619 | | | 0.0001 | | | 4.8 | | | | | 18.2 | | 0.626446 |
| 11A | | 16.816 | | | HAU2681-366/0 | | | | 27.854 | | | | 0.0001 | | | 7.18 | | | 26 | | 1.14333 | | | 23.258 | | | 0.0001 | | | 5.73 | | | | | 21.3 | | 0.678387 |
| 11A | | 17.167 | | | NAU3770-187/195 | | | | 30.491 | | | | 0.0001 | | | 8.24 | | | 29.2 | | 1.2142 | | | 26.362 | | | 0.0001 | | | 6.49 | | | | | 23.8 | | 0.717721 |
| 11A | | 17.249 | | | MUCS088-170/162 | | | | 33.482 | | | | 0.0001 | | | 9.6 | | | 33.1 | | 1.29048 | | | 28.879 | | | 0.0001 | | | 7.27 | | | | | 26.3 | | 0.752654 |
| 11A | | 17.72 | | | CIR196-205/206 | | | | 31.602 | | | | 0.0001 | | | 8.9 | | | 31.3 | | 1.25712 | | | 30.281 | | | 0.0001 | | | 7.36 | | | | | 26.7 | | 0.759408 |
| 11A | | 18.303 | | | NAU1232--205/208 | | | | 18.945 | | | | 0.0001 | | | 6.57 | | | 24.6 | | 1.11567 | | | 16.884 | | | 0.0001 | | | 5.23 | | | | | 19.8 | | 0.656401 |
| 11A | | 18.821 | | | MGhES-16-0/221 | | | | 33.07 | | | | 0.0001 | | | 9.26 | | | 32.2 | | 1.27722 | | | 29.778 | | | 0.0001 | | | 7.16 | | | | | 25.9 | | 0.750654 |
| 11A | | 19.337 | | | NAU2016-0/247 | | | | 29.823 | | | | 0.0001 | | | 8.41 | | | 29.7 | | 1.23024 | | | 26.543 | | | 0.0001 | | | 6.5 | | | | | 23.8 | | 0.721428 |
| 11A | | 20.553 | | | NAU5428-236/0 | | | | 25.711 | | | | 0.0001 | | | 6.36 | | | 23.4 | | 1.08298 | | | 21.364 | | | 0.0001 | | | 5.52 | | | | | 20.6 | | 0.666254 |
| 11A | | 22.411 | | | NAU3390-213/206 | | | | 19.244 | | | | 0.0001 | | | 5.54 | | | 20.7 | | 1.03375 | | | 13.317 | | | 0.0005 | | | 3.62 | | | | | 14.1 | | 0.558098 |
| 11A | | 23.058 | | | BNL1231-208/217 | | | | 21.219 | | | | 0.0001 | | | 5.26 | | | 20 | | 1.01434 | | | 15.589 | | | 0.0001 | | | 3.55 | | | | | 13.9 | | 0.553955 |
| 11A | | 23.294 | | | NAU2152-221/215 | | | | 19.699 | | | | 0.0001 | | | 5.03 | | | 19 | | 0.993501 | | | 14.025 | | | 0.0005 | | | 3.32 | | | | | 13 | | 0.537206 |
| 11A | | 25.798 | | | Gh288-173/189 | | | | 18.458 | | | | 0.0001 | | | 3.9 | | | 15.1 | | 0.872215 | | | 14.385 | | | 0.0005 | | | 2.63 | | | | | 10.4 | | 0.474783 |
| 11A | | 26.515 | | | CIR003-149/147 | | | | 13.94 | | | | 0.0005 | | | 3.38 | | | 13.2 | | 0.814794 | | | 9.46 | | | 0.005 | | | 2.39 | | | | | 9.5 | | 0.453462 |
| 11A | | 26.873 | | | MUSB0404-320/328 | | | | 16.601 | | | | 0.0001 | | | 3.93 | | | 15.2 | | 0.872631 | | | 12.591 | | | 0.0005 | | | 2.82 | | | | | 11.1 | | 0.48943 |
| 11A | | 30.156 | | | BNL4011-175/0 | | | | 9.954 | | | | 0.005 | | | 2.21 | | | 8.9 | | 0.665233 | | | 7.082 | | | 0.01 | | | 1.49 | | | | | 6.1 | | 0.360134 |
| 11A | | 38.395 | | | NAU5428-0/234 | | | | 12.425 | | | | 0.0005 | | | 2.31 | | | 9.2 | | 0.897908 | | | 12.162 | | | 0.0005 | | | 2.22 | | | | | 8.9 | | 0.577996 |
| 11A | | 39.94 | | | MUSB0404-0/314 | | | | 8.249 | | | | 0.005 | | | 1.81 | | | 7.3 | | 0.606976 | | | 10.021 | | | 0.005 | | | 2.23 | | | | | 8.9 | | 0.438873 |
| 3 | | 0 | | | MUSB0639-335/337 | | | | 4.468 | | | | 0.05 | | | 1.26 | | | 5.2 | | 0.486228 | | | 5.27 | | | 0.05 | | | 1.08 | | | | | 4.4 | | 0.307247 |
| 3 | | 1.354 | | | BNL226-231/248 | | | | 4.144 | | | | 0.05 | | | 1.19 | | | 4.9 | | 0.472558 | | | 4.243 | | | 0.05 | | | 1.01 | | | | | 4.2 | | 0.297972 |
| 3 | | 10.5 | | | BNL3792-540/0 | | | | 7.727 | | | | 0.005 | | | 1.93 | | | 7.7 | | 0.595899 | | | 6.918 | | | 0.01 | | | 1.49 | | | | | 6.1 | | 0.359863 |
| 3 | | 16.91 | | | Gh109-685/0 | | | | 5.652 | | | | 0.05 | | | 1.1 | | | 4.5 | | 0.455 | | | 4.515 | | | 0.05 | | | 1.09 | | | | | 4.5 | | 0.3108 |
| 3 | | 27.132 | | | BNL3257-375/0 | | | | 6.480 | | | | 0.01 | | | 1.45 | | | 5.9 | | 0.523858 | | | 3.991 | | | 0.05 | | | 0.89 | | | | | 3.7 | | 0.282413 |
| 3 | | 47.574 | | | MUSS396-0/300 | | | | 9.279 | | | | 0.01 | | | 1.92 | | | 7.7 | | 0.617857 | | | 9.195 | | | 0.005 | | | 2.11 | | | | | 8.5 | | 0.442282 |
| 1A | | 44.118 | | | NAU4045-185/195 | | | | 8.893 | | | | 0.005 | | | 1.66 | | | 6.8 | | -0.56758 | | | 8.749 | | | 0.005 | | | 2.42 | | | | | 9.8 | | -0.46518 |
| 1. **Testcross NemX x F_1_ (Pima S-7 x SJ-2)** | | | | | | | | | | | | | | | | | | | | | | | | | | | | | | | | | | | | | |
|  |  | |  | | | | GI | | | | | | | | | | | | | | | LogEGR | | | | | | | | | | | | | | | |
|  |  | |  | | | | K* test | | | | | | | IM | | | | | | | | K* test | | | | | | | IM | | | | | | | | |
| Chr | Position | | Locus | | | | K* | | | Signif. | | | | LOD | | | | % Expl. | | Additive | | K* | | | | Signif. | | | LOD | | | | % Expl. | | | Additive | |
| 11-1 | 0 | | HAU1283-155/0 | | | | 23.659 | | | 0.0001 | | | | 9.02 | | | | 55.7 | | -1.39846 | | 27.543 | | | | 0.0001 | | | 7.3 | | | | 48.3 | | | -0.68248 | |
| 11-1 | 0 | | HAU1809-283/0 | | | | 23.659 | | | 0.0001 | | | | 9.02 | | | | 55.7 | | -1.39846 | | 27.543 | | | | 0.0001 | | | 7.3 | | | | 48.3 | | | -0.68248 | |
| 11-1 | 0 | | CIR112-251/0 | | | | 23.659 | | | 0.0001 | | | | 9.02 | | | | 55.7 | | -1.39846 | | 27.543 | | | | 0.0001 | | | 7.3 | | | | 48.3 | | | -0.68248 | |
| 11-1 | 1.585 | | BNL1231-208/210 | | | | 24.391 | | | 0.0001 | | | | 8.34 | | | | 52.9 | | -1.36516 | | 25.489 | | | | 0.0001 | | | 6.28 | | | | 43.3 | | | -0.64732 | |
| 11-1 | 1.95 | | CIR316-215/210 | | | | 25.854 | | | 0.0001 | | | | 9.42 | | | | 57.3 | | -1.41808 | | 26.563 | | | | 0.0001 | | | 6.93 | | | | 46.5 | | | -0.66992 | |
| 11-1 | 1.95 | | CT49-263/291 | | | | 25.854 | | | 0.0001 | | | | 9.42 | | | | 57.3 | | -1.41808 | | 26.563 | | | | 0.0001 | | | 6.93 | | | | 46.5 | | | -0.66992 | |
| 11-1 | 1.95 | | NAU5428-236/0 | | | | 25.854 | | | 0.0001 | | | | 9.42 | | | | 57.3 | | -1.41808 | | 26.563 | | | | 0.0001 | | | 6.93 | | | | 46.5 | | | -0.66992 | |
| 11-1 | 1.95 | | CM140-117/0 | | | | 24.96 | | | 0.0001 | | | | 9.42 | | | | 57.3 | | -1.41808 | | 25.594 | | | | 0.0001 | | | 6.93 | | | | 46.5 | | | -0.66992 | |
| 11-1 | 1.95 | | HAU2681-366/0 | | | | 25.854 | | | 0.0001 | | | | 9.42 | | | | 57.3 | | -1.41808 | | 26.563 | | | | 0.0001 | | | 6.93 | | | | 46.5 | | | -0.66992 | |
| 11-1 | 1.95 | | NAU3770-187/0 | | | | 25.854 | | | 0.0001 | | | | 9.42 | | | | 57.3 | | -1.41808 | | 26.563 | | | | 0.0001 | | | 6.93 | | | | 46.5 | | | -0.66992 | |
| 11-1 | 1.95 | | CIR196-205/0 | | | | 25.854 | | | 0.0001 | | | | 9.42 | | | | 57.3 | | -1.41808 | | 26.563 | | | | 0.0001 | | | 6.93 | | | | 46.5 | | | -0.66992 | |
| 11-1 | 1.95 | | MUCS088-170/0 | | | | 25.854 | | | 0.0001 | | | | 9.42 | | | | 57.3 | | -1.41808 | | 26.563 | | | | 0.0001 | | | 6.93 | | | | 46.5 | | | -0.66992 | |
| 11-1 | 1.95 | | NAU1232-205/0 | | | | 25.854 | | | 0.0001 | | | | 9.42 | | | | 57.3 | | -1.41808 | | 26.563 | | | | 0.0001 | | | 6.93 | | | | 46.5 | | | -0.66992 | |
| 11-1 | 1.95 | | NAU2152-221/0 | | | | 25.854 | | | 0.0001 | | | | 9.42 | | | | 57.3 | | -1.41808 | | 26.563 | | | | 0.0001 | | | 6.93 | | | | 46.5 | | | -0.66992 | |
| 11-1 | 2.089 | | NAU3390-213/0 | | | | 25.854 | | | 0.0001 | | | | 9.42 | | | | 57.3 | | -1.41808 | | 26.563 | | | | 0.0001 | | | 6.93 | | | | 46.5 | | | -0.66992 | |
| 11-1 | 2.315 | | CIR069-271/0 | | | | 27.523 | | | 0.0001 | | | | 10.86 | | | | 62.5 | | -1.48322 | | 29.238 | | | | 0.0001 | | | 7.75 | | | | 50.3 | | | -0.69808 | |
| 11-1 | 3.901 | | Gh288-173/0 | | | | 22.826 | | | 0.0001 | | | | 7.28 | | | | 48.2 | | -1.30038 | | 23.182 | | | | 0.0001 | | | 5.8 | | | | 40.8 | | | -0.62716 | |
| 11-1 | 3.901 | | CIR003-149/0 | | | | 22.826 | | | 0.0001 | | | | 7.28 | | | | 48.2 | | -1.30038 | | 23.182 | | | | 0.0001 | | | 5.8 | | | | 40.8 | | | -0.62716 | |
| 1. **F_2_ (Pima S-7 x SJ-2)** | | | | | | | | | | | | | | | | | | | | | | | | | | | | | | | | | | | | | |
|  |  | |  | | | GI | | | | | | | | | | | | | | | | | LogEGR | | | | | | | | | | | | | | |
|  |  | |  | | | K* test | | | | | | IM | | | | | | | | | | | K* test | | | | | IM | | | | | | | | | |
| chr | position | | locus | | | K* | | | Signif | | | LOD | | | % expl | | Additive | | | Dominance | | | K* | | Signif | | | LOD | | | | % expl | | Additive | | | Dominance |
| 11 | 0 | | HAU1283-149/155 | | | 8.293 | | | 0.05 | | | 1.95 | | | 8.8 | | -0.76551 | | | -0.3833 | | | 9.627 | | 0.01 | | | 2.35 | | | | 10.5 | | -0.21927 | | | -0.04362 |
| 11 | 13.492 | | CIR316-215/210 | | | 15.722 | | | 0.0005 | | | 3.49 | | | 15 | | -1.05808 | | | -0.00359 | | | 22.785 | | 0.0001 | | | 5.8 | | | | 23.7 | | -0.32939 | | | 0.046964 |
| 11 | 20.254 | | CIR069-271/267 | | | 6.779 | | | 0.05 | | | 1.47 | | | 6.7 | | -0.74388 | | | -0.22286 | | | 16.57 | | 0.0005 | | | 3.51 | | | | 15.2 | | -0.28473 | | | -0.0195 |
| 11 | 27.367 | | MUSB1076-296/306 | | | 5.094 | | | 0.1 | | | 1.13 | | | 5.2 | | -0.62096 | | | -0.01006 | | | 11.283 | | 0.005 | | | 2.32 | | | | 10.3 | | -0.21808 | | | 5.24E-05 |
| 11 | 36.392 | | CIR196-205/206 | | | 3.737 | | | - | | | 1.05 | | | 4.9 | | -0.64999 | | | 0.00937 | | | 6.708 | | 0.05 | | | 1.56 | | | | 7.1 | | -0.19023 | | | 0.068412 |
| 11 | 38.475 | | MUCS088-170/162 | | | 6.087 | | | 0.05 | | | 1.76 | | | 8.1 | | -0.82331 | | | -0.04036 | | | 7.886 | | 0.05 | | | 2.71 | | | | 12.1 | | -0.24317 | | | 0.098963 |
| 1. **F_2:8_ (NemX x SJ-2)** | | | | | | | | | | | | | | | | | | | | | | | | | | | | | | | | | | | | | |
|  | |  | |  | | | | GI | | | | | | | | | | | | | | | | LogEGR | | | | | | | | | | | | | |
|  | |  | |  | | | | K* test | | | | | | | | IM | | | | | | | | K* test | | | | | | | IM | | | | | | |
| Chr | | Position | | Locus | | | | K* | | | Signif. | | | | | LOD | | | % Expl. | | Additive | | | K* | | | Signif. | | | | LOD | | | | % Expl. | | Additive |
| 11 | | 0 | | UCR91-236/0 | | | | 22.267 | | | 0.0001 | | | | | 5.94 | | | 32.7 | | -1.62723 | | | 18.242 | | | 0.0001 | | | | 5.05 | | | | 28.6 | | -0.68061 |
| 11 | | 14.183 | | UCR56-236/235 | | | | 32.219 | | | 0.0001 | | | | | 13.32 | | | 58.9 | | -2.0101 | | | 33.596 | | | 0.0001 | | | | 12.3 | | | | 56 | | -0.87708 |
| 11 | | 14.947 | | UCR49-292/290 | | | | 36.572 | | | 0.0001 | | | | | 15.82 | | | 65.2 | | -2.11366 | | | 36.088 | | | 0.0001 | | | | 13.8 | | | | 60.2 | | -0.90844 |
| 11 | | 15.238 | | UCR90-199/0 | | | | 38.493 | | | 0.0001 | | | | | 17.81 | | | 69.5 | | -2.18434 | | | 37.021 | | | 0.0001 | | | | 15.11 | | | | 63.5 | | -0.93397 |
| 11 | | 15.838 | | CIR316-221/210 | | | | 41.262 | | | 0.0001 | | | | | 19.07 | | | 72 | | -2.22059 | | | 40.851 | | | 0.0001 | | | | 16.34 | | | | 66.4 | | -0.95409 |
| 11 | | 16.532 | | GHACC1-300/0 | | | | 47.476 | | | 0.0001 | | | | | 25.76 | | | 82.1 | | -2.37311 | | | 44.37 | | | 0.0001 | | | | 20.24 | | | | 74.1 | | -1.00884 |
| 11 | | 16.532 | | UCR61-202/0 | | | | 47.476 | | | 0.0001 | | | | | 25.76 | | | 82.1 | | -2.37311 | | | 44.37 | | | 0.0001 | | | | 20.24 | | | | 74.1 | | -1.00884 |
| 11 | | 16.532 | | UCR91-240/0 | | | | 47.476 | | | 0.0001 | | | | | 25.76 | | | 82.1 | | -2.37311 | | | 44.37 | | | 0.0001 | | | | 20.24 | | | | 74.1 | | -1.00884 |
| 11 | | 18.011 | | M9E2-327/0 | | | | 42.747 | | | 0.0001 | | | | | 23.35 | | | 79 | | -2.33829 | | | 45.149 | | | 0.0001 | | | | 25.37 | | | | 81.7 | | -1.0635 |
| 11 | | 20.225 | | M5E1-0/545 | | | | 31.038 | | | 0.0001 | | | | | 12.13 | | | 55.6 | | -1.95221 | | | 35.202 | | | 0.0001 | | | | 13.74 | | | | 60.2 | | -0.90878 |
| 11 | | 22.669 | | M4E5-258/0 | | | | 23.669 | | | 0.0001 | | | | | 8.89 | | | 44.9 | | -1.76968 | | | 30.658 | | | 0.0001 | | | | 10.94 | | | | 52.1 | | -0.85274 |
| 11 | | 23.501 | | M4E5-0/257 | | | | 22.03 | | | 0.0001 | | | | | 7.94 | | | 41.2 | | -1.70441 | | | 28.446 | | | 0.0001 | | | | 9.79 | | | | 48.3 | | -0.82468 |
| 11 | | 32.924 | | BNL1231-217/210 | | | | 13.779 | | | 0.0005 | | | | | 4.2 | | | 24.4 | | -1.35833 | | | 6.967 | | | 0.01 | | | | 2.77 | | | | 16.9 | | -0.50521 |
| 11 | | 35.414 | | Gh288-192/189 | | | | 13.272 | | | 0.0005 | | | | | 4.19 | | | 24.5 | | -1.35804 | | | 5.358 | | | 0.01 | | | | 2.16 | | | | 13.5 | | -0.45102 |
| 1. **F_2_ (NemX x SJ-2)** | | | | | | | | | | | | | | | | | | | | | | | | | | | | | | | | | | | | | |
|  |  | |  | | | GI | | | | | | | | | | | | | | | | | LogEGR | | | | | | | | | | | | | | |
|  |  | |  | | | K* test | | | | | | IM | | | | | | | | | | | K* test | | | | | IM | | | | | | | | | |
| chr | position | | locus | | | K* | | | Signif | | | LOD | | | % expl | | Additive | | | Dominance | | | K* | | Signif | | | LOD | | | | % expl | | Additive | | | Dominance |
| 11 | 0 | | UCR91-240/0 | | | 19.394 | | | 0.0001 | | | 9.08 | | | 36.5 | | -0.93634 | | | 0.129415 | | | 20.555 | | 0.0001 | | | 5.66 | | | | 25 | | -0.52936 | | | 0.11988 |
| 11 | 1.428 | | CIR316-221/210 | | | 33.627 | | | 0.0001 | | | 9.24 | | | 35.8 | | -0.95977 | | | 0.084309 | | | 26.501 | | 0.0001 | | | 5.76 | | | | 24.1 | | -0.53658 | | | 0.077298 |
| 11 | 3.422 | | UCR61-202/0 | | | 17.78 | | | 0.0001 | | | 9.08 | | | 36.3 | | -0.96145 | | | 0.143061 | | | 21.65 | | 0.0001 | | | 7.19 | | | | 32.1 | | -0.60497 | | | 0.186648 |
| 11 | 4.061 | | GHACC1-300/0 | | | 18.639 | | | 0.0001 | | | 9.3 | | | 37.2 | | -0.98534 | | | 0.116707 | | | 23.455 | | 0.0001 | | | 7.52 | | | | 33.2 | | -0.62234 | | | 0.179116 |
| 11 | 4.615 | | UCR90-199/0 | | | 20.453 | | | 0.0001 | | | 8.82 | | | 35.9 | | -0.95474 | | | 0.152933 | | | 23.813 | | 0.0001 | | | 7.45 | | | | 32.8 | | -0.61179 | | | 0.196437 |
| 11 | 6.965 | | M9E2-327/0 | | | 14.894 | | | 0.0001 | | | 7.96 | | | 33.4 | | -0.95677 | | | 0.142088 | | | 16.079 | | 0.0001 | | | 6.87 | | | | 29.9 | | -0.59092 | | | 0.241085 |
| 11 | 8.488 | | UCR49-292/290 | | | 28.061 | | | 0.0001 | | | 7.89 | | | 31.6 | | -0.9029 | | | -0.011140 | | | 27.222 | | 0.0001 | | | 6.88 | | | | 28.2 | | -0.56827 | | | 0.176342 |
| 11 | 11.894 | | M5E1-0/545 | | | 14.792 | | | 0.0001 | | | 6.68 | | | 30.1 | | -0.8881 | | | -0.187153 | | | 16.792 | | 0.0001 | | | 7.22 | | | | 32.1 | | -0.64277 | | | 0.063293 |
| 11 | 13.654 | | M4E5-0/257 | | | 14.833 | | | 0.0001 | | | 6.64 | | | 31 | | -0.90336 | | | -0.175151 | | | 12.464 | | 0.0001 | | | 6.5 | | | | 29.9 | | -0.62166 | | | 0.00717 |
| 11 | 25.728 | | BNL1231-217/210 | | | 9.518 | | | 0.01 | | | 2.18 | | | 9.9 | | -0.51175 | | | 0.122671 | | | 19.546 | | 0.0001 | | | 3.56 | | | | 15.7 | | -0.44257 | | | 0.014127 |
| 11 | 29.424 | | Gh288-192/189 | | | 8.67 | | | 0.05 | | | 1.85 | | | 8.5 | | -0.47643 | | | 0.0564286 | | | 18.61 | | 0.0001 | | | 3.03 | | | | 13.5 | | -0.40322 | | | -0.07818 |
| 11 | 41.367 | | DPL209-220/230-231 | | | 4.84 | | | 0.1 | | | 1.22 | | | 5.7 | | -0.35737 | | | -0.136357 | | | 10.363 | | 0.01 | | | 2.09 | | | | 9.5 | | -0.31851 | | | -0.11252 |

% expl: percentage of phenotypic variance explained.

K*: Kruskal-Wallis analysis test regraded as the nonparametric equivalent of the one-way analysis of variance.
